# Ferroptosis: a new mechanism of traditional Chinese medicine for treating ulcerative colitis

**DOI:** 10.3389/fphar.2024.1379058

**Published:** 2024-06-04

**Authors:** Yingyi Wang, Yanwei Hao, Lingling Yuan, Huaie Tian, Xuhui Sun, Yi Zhang

**Affiliations:** ^1^ Department of Gastroenterology, Hospital of Chengdu University of Traditional Chinese Medicine, Chengdu, China; ^2^ Department of Geriatrics, Hospital of Chengdu University of Traditional Chinese Medicine, Chengdu, China

**Keywords:** ferroptosis, traditional Chinese medicine, ulcerative colitis, Glutathion (GSH), glutathione peroxidase 4 (GPX4)

## Abstract

Ulcerative colitis (UC), a subtype of inflammatory bowel disease, manifests with symptoms such as abdominal pain, diarrhea, and mucopurulent, bloody stools. The pathogenesis of UC is not fully understood. At present, the incidence of UC has increased significantly around the world. Conventional therapeutic arsenals are relatively limited, with often poor efficacy and many adverse effects. In contrast, traditional Chinese medicine (TCM) holds promise due to their notable effectiveness, reduced recurrence rates, and minimal side effects. In recent years, significant progress has been made in the basic research on TCM for UC treatment. It has been found that the inhibition of ferroptosis through the intervention of TCM can significantly promote intestinal mucosal healing and reverse UC. The mechanism of action involves multiple targets and pathways.

**Aim of the review:**

This review summarizes the experimental studies on the targeted regulation of ferroptosis by TCM and its impact on UC in recent years, aiming to provide theoretical basis for the prevention, treatment, and further drug development for UC.

**Results:**

Ferroptosis disrupts antioxidant mechanisms in intestinal epithelial cells, damages the intestinal mucosa, and participates in the pathological process of UC. TCM acts on various pathways such as *Nrf2*/*HO-1* and GSH/GPX4, blocking the pathological progression of ferroptosis in intestinal epithelial cells, inhibiting pathological damage to the intestinal mucosa, and thereby alleviating UC.

**Conclusion:**

The diverse array of TCM single herbs, extracts and herbal formulas facilitates selective and innovative research and development of new TCM methods for targeting UC treatment. Although progress has been made in studying TCM compound formulas, single herbs, and extracts, there are still many issues in clinical and basic experimental designs, necessitating further in-depth scientific exploration and research.

## Graphical Abstract

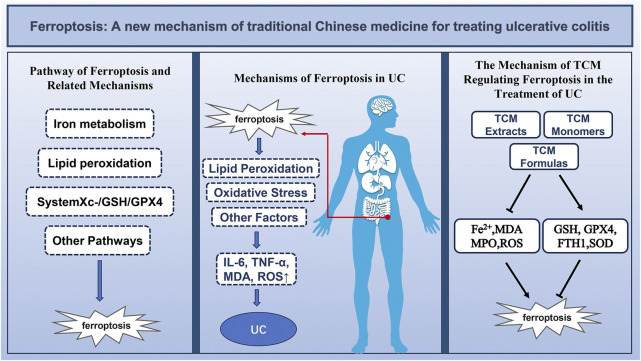

## 1 Introduction

UC is a prevalent chronic inflammatory bowel disease characterized by clinical manifestations such as abdominal pain, diarrhea, and mucopurulent bloody stools, often involving the entire colon and posing a significant risk for colon cancer (Le Berre et al., 2019; Le Berre et al., 2020). UC is an incurable lifelong disease with a relatively low mortality rate, but a high incidence. And its epidemiological trend is continuously evolving, making it a global health challenge (Lophaven et al., 2017; Ng et al., 2017). According to data from The Lancet, the global incidence of UC is approximately 5 million people in 2023 (Le Berre et al., 2023). Surveys conducted by the University of Calgary indicate that in the 21st century, the incidence and hospitalization rates of UC in emerging industrialized countries are rapidly increasing (Buie et al., 2023), which greatly predisposes individuals to colorectal cancer (Paik, 2022). Despite extensive research, the precise etiology of UC remains elusive, although it is widely believed to be associated with immune responses, oxidative stress, and inflammatory reactions (Le Berre et al., 2023). Current pharmacological interventions for UC primarily include salicylates, glucocorticoids, and biologics. Some patients require colectomy to treat UC. However, these treatments are plagued by substantial side effects and a propensity for complications. In comparison, TCM often adopts a comprehensive approach, combining multiple methods such as oral administration of Chinese herbal medicine, herbal enemas, acupuncture, and other aspects to exert multifaceted effects. TCM, with its holistic concept and the characteristics of syndrome differentiation for treatment, can utilize the interactions among various Chinese herbal medicines to treat UC. This can help improve patients’ quality of life and reduce the side effects of Western medical treatments (Wang et al., 2023). The composite morbidity of UC continues to rise, placing a burden on the global healthcare system.Consequently, elucidating the mechanisms underlying UC and identifying more effective and stable therapeutic strategies have become imperative. The destruction of the intestinal barrier and loss of the mucosa form the basis of UC. The intestinal mucosal barrier is provided by mucus secreted by goblet cells in the intestinal epithelial cells (IEC), and excessive death of these goblet cells is an important cause of UC onset (Leppkes and Neurath, 2020). Recently, with advances in research, several types of cell death, including autophagy and ferroptosis, have been confirmed to be associated with UC, in addition to apoptosis and necrosis (Dong et al., 2021).

Ferroptosis is a form of cell death characterized by iron deposition and iron-dependent lipid peroxidation. It differs morphologically, biochemically, and genetically from apoptosis, necroptosis, and autophagy (Dixon et al., 2012). The occurrence of ferroptosis is attributed to the accumulation of excess lipid reactive oxygen species (ROS), leading to the peroxidation of polyunsaturated fatty acids (PUFA) and membrane damage (Dixon et al., 2012). In recent years, numerous studies have shown a close association between ferroptosis and UC (Xu et al., 2020; Zhang et al., 2023). Research has observed signs of ferroptosis, including iron deposition, accumulation of lipid peroxides, depletion of glutathione (GSH), and inactivation of Glutathione peroxidase 4 (GPX4), during the onset of UC in mouse models (Dong et al., 2021). Additionally, dysfunction in key ferroptosis regulatory systems (such as GPX4, GSH) in mouse models can lead to the worsening of UC (Luo et al., 2023). Furthermore, iron inhibitors have demonstrated intestinal protective effects in various UC experimental models (Huang et al., 2022). Therefore, ferroptosis represents a promising new target for the treatment of UC.

Traditional Chinese Medicine (TCM) has a long history of use worldwide for improving human health due to its natural origins and efficacy. TCM treatments for chronic diseases are characterized by multiple metabolites, targets, and pathways (Feng et al., 2022). This is demonstrated in the treatment of UC by the following: a single Chinese herbal medicine may contain multiple metabolites, each with different pathways for treating UC. For example, berberine extracted from Coptis chinensis can treat UC by regulating pathways such as NK-κB, PI3K-AKT, MAPK, and *Nrf2* (Wang et al., 2021). Chinese herbal medicines can be combined into herbal formulas that interact with each other, such as Shaoyao Gancao Tang involving multiple targets like GSH and MDA (Hu et al., 2023). In the prevention and treatment of UC, TCM offers clinical advantages over Western medicine, including fewer complications, minimal side effects, and lower recurrence rates (Liu et al., 2022). Records of TCM prescriptions for treating UC date back to ancient times, such as “Bai Tou Weng Tang (Liu et al., 2023)” and “Huang Qin Tang (Jiang et al., 2023)” in “Treatise on Febrile Diseases”. Modern pharmacological studies have demonstrated that TCM can prevent and treat UC through various mechanisms, such as reducing oxidative stress, regulating autophagy, and exerting anti-inflammatory effects. Among these mechanisms, TCM targeting ferroptosis has shown significant efficacy (Chen et al., 2022).

Traditional Chinese medicine believes that dampness-heat is the main pathogenesis of UC. “Dampness-heat” and “heat-toxins” are believed to accumulate in the intestines, leading to disorders in intestinal obstruction, stagnation of qi and blood, damage to the intestinal collaterals, and causing dysentery with red and white pus and blood. The essence of ferroptosis, namely, the accumulation of iron deposition and excessive lipid peroxidation products, is a microscopic manifestation of what is described as “dampness-heat” in traditional Chinese medicine. Many animal experiments have shown that traditional Chinese medicines used under the guidance of theories such as “clearing heat and removing dampness” can effectively reduce the severity of ferroptosis. This demonstrates the close connection between ferroptosis and “dampness-heat.” Although ferroptosis protection may occur in any plant extract, it is still necessary to explore the effects of single Chinese herbal medicines and Chinese herbal compound prescriptions on ferroptosis under the guidance of traditional Chinese medicine theory. This is to seek a closer connection between traditional Chinese medicine and modern pharmacology in order to bring better treatment options for UC.

Therefore, this paper systematically reviews the mechanisms of ferroptosis and its role in UC. Moreover, we summarize the current evidence supporting the use of TCM to target ferroptosis for improving UC, aiming to provide a new theoretical perspective for future research on the potential roles of TCM compounds and formulas with similar capabilities to target ferroptosis in the treatment of UC.

## 2 Pathway of ferroptosis and related mechanisms

### 2.1 Pathway of ferroptosis

Ferroptosis discovered by Dolma in 2003 initially (Dolma et al., 2003), was formally named in 2012 (Dixon et al., 2012). Ferroptosis refers to a form of programmed cell death characterized by the excessive iron-catalyzed formation of ROS, which promotes lipid peroxidation, disrupts cell membranes, and leads to cell death (Dixon et al., 2012). Its key features include the accumulation of iron ions and iron-dependent lipid peroxidation (Xie et al., 2016).

Lipid peroxidation refers to the process in which PUFAs on the lipid membrane are oxidized by ROS, leading to the generation of lipid peroxides, which can disrupt the membrane and cause cell death (Tang and Kroemer, 2020). ROS encompass oxygen-related metabolites, oxygen-free radicals, and easily formed free radicals in organisms, including superoxide anion (O^2-^), hydrogen peroxide (H_2_O_2_), hydroxyl radicals (-OH), and lipid peroxides (L-OOH), among others (Su et al., 2019). Under normal circumstances, the highly reactive free radical O^2-^ generated by the mitochondrial respiratory chain is converted into less active H_2_O_2_ by superoxide dismutase (SOD), and subsequently cleared by GPX4, catalase, and peroxiredoxin (Mailloux, 2015). However, in the presence of excess iron within the cell, H_2_O_2_ triggers the Fenton reaction under the catalysis of ferrous iron (Fe^2+^), leading to the production of -OH (Shi et al., 2023). The Fenton reaction involves the oxidation-reduction reaction of H_2_O_2_ with Fe^2+^, generating -OH, iron (Fe^3+^), and hydroxide (OH-) (Shi et al., 2023). Unstable -OH and OH- combine with free lipids within the cell to generate lipid-toxic ROS, including lipid peroxyl radicals (L-OO-) and L-OOH (Shi et al., 2023), with the catalytic action of arachidonate lipoxygenases (ALOXs) and cytochrome P450 reductases (PORs) (Stoyanovsky et al., 2019). The accumulation of these lipid peroxides can lead to cell death through several mechanisms: 1) disruption and pore formation in the membrane causing loss of ion balance; 2) changes in membrane composition altering interactions with embedded proteins; 3) oxidative cleavage of PUFAs releasing ROS that interfere with other cellular processes (Agmon and Stockwell, 2017).

The detailed process outlined above illustrates how iron deposition leads to lipid peroxidation and subsequent cell death. However, the downstream pathways of ferroptosis are not yet fully understood. It is currently believed that the ultimate result of lipid peroxidation is the disruption of PUFA metabolism and membrane homeostasis, ultimately leading to cell death.

**FIGURE 1:**
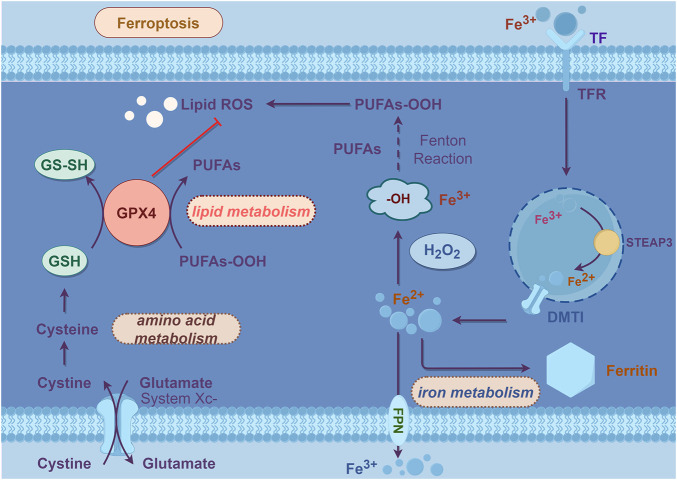
Mechanisms of ferroptosis.

The mechanism of ferroptosis is illustrated in Figure 1 in line.

### 2.2 Mechanisms of ferroptosis

#### 2.2.1 Iron metabolism

Iron, being an essential trace element in the human body, plays a critical role in maintaining cellular homeostasis (Rishi et al., 2015). The iron metabolism process is primarily regulated by iron-related genes, including heat shock protein beta 1 (HSPB1), iron-regulatory protein 2 (IRP2), transferrin receptor 2 (TFR2), etc., (Li et al., 2019). When iron ions enter the cell, Fe^2+^ is oxidized to Fe^3+^ by hephaestin (HEPH) and binds to transferrin (TF). This complex is then taken up by the cell through transferrin receptor 1 (TFR1) and subsequently reduced to Fe^2+^ by metalloreductase 3 (STEAP3). The released Fe^2+^ enters the cytoplasm through divalent metal transporter 1 (DMT1). Excess Fe^2+^ will be oxidized into Fe^3+^ through the ferritin heavy chain and stored in a stable form in ferritin (Wang et al., 2023).

Iron storage protein ferritin, comprising ferritin light chain (FTL) and ferritin heavy chain 1 (FTH1), plays a crucial role in storing 70%–80% of newly absorbed iron within cells (Zhang et al., 2021). Functioning as the most significant iron storage complex in cells, ferritin modulates iron levels by either storing or releasing excess iron, thereby regulating iron overload or deficiency (Santana-Codina et al., 2021). Recent researches have elucidated the process of iron release from ferritin, termed ferritinophagy (Bengson et al., 2023). Experimental findings have demonstrated that under conditions of low cellular iron levels, ferritin undergoes degradation via ferritinophagy, leading to the release of ferric ions, subsequently converted into ferrous ions (Nai et al., 2021). This mechanism is closely associated with a receptor known as NCOA4, which binds to ferritin and facilitates its delivery to autophagosomes and lysosomes for degradation. NCOA4-mediated ferritinophagy serves to augment cellular iron content, thereby promoting ferroptosis (Hou et al., 2016). Numerous studies have substantiated the critical involvement of NCOA4-mediated ferritinophagy in the pathogenesis of various diseases, including neurodegenerative disorders, metabolic ailments, infectious conditions, and cancer (Ajoolabady et al., 2021).

#### 2.2.2 Lipid peroxidation

As previously mentioned, abnormal iron metabolism leads to lipid peroxidation, causing cellular ferroptosis. PUFAs are the main targets of lipid peroxidation (Blaha et al., 2000). PUFAs can be acylated into polyunsaturated fatty acid chains in phospholipids (PUFA-PL), participating in the composition of cell membranes. The process of PUFAs activation and incorporation into membrane phospholipids is related to acyl-CoA synthetase long chain family member 4 (ACSL4) and lysophosphatidylcholine acyltransferase 3 (LPCAT3). Under normal conditions, ACSL4 catalyzes the reaction of PUFA with coenzyme A (CoA) to produce PUFA-CoA derivatives, which then esterify into PUFA-PL. Subsequently, LPCAT3 selectively inserts acyl groups into phosphatidylcholine, synthesizing PUFA-PL, completing the metabolism of cell membranes. During ferroptosis, esterified PUFAs integrated into the cell membrane will initiate lipid autoxidation, damaging the cell membrane (Dixon et al., 2015).

Extensive data indicate that ACSL4 and LPCAT3 play important roles in inducing ferroptosis (Doll et al., 2017). Under the catalysis of ACSL4 and LPCAT3, the accumulation of PL-PUFA-OOH on the cell membrane induces ferroptosis. Their absence or inactivation inhibits ferroptosis.

Exogenous peroxidation-reactive nonconjugated PUFAs, such as phosphatidylethanolamine containing arachidonic acid (AA) also are considered a key triggering pathway for ferroptosis (Hirata et al., 2023). PUFA-PL containing AA or adrenic acid are oxidized to phospholipid hydroperoxides (PL-PUFA-OOH) through the Fenton reaction, thereby driving the process of ferroptosis (Do and Xu, 2023).

The LOXs family is crucial for initiating enzymatic lipid peroxidation in ferroptosis. For example, ALOX5 (arachidonate lipoxygenase 5) and ALOX12 (arachidonate lipoxygenase 12) encoded by LOX5 and LOX12 can help induce ferroptosis sensitivity by converting esterified PUFAs into lipid hydroperoxides (Hassannia et al., 2019). In addition to LOXs, NADPH-cytochrome P450 reductase (POR), located in the endoplasmic reticulum, also participates in the enzymatic lipid peroxidation process to regulate ferroptosis (Zou et al., 2020).

#### 2.2.3 SystemXc-/GSH/GPX4

The antioxidant axis systemXc-/GSH/GPX4 is the key to regulate ferroptosis. 2014, YANG and colleagues discovered that erastin depletes GSH to promote ROS formation and induce ferroptosis (Yang et al., 2014). Further researches revealed that GPX4 and the reduced and oxidized forms of GSH constitute a major cellular antioxidant system, which contains transmembrane proteins such as solute carrier family 7 member 11 (SLC7A11) and solute carrier family 3 member 2 (SLC3A2). It can eliminate ROS generated during intracellular metabolism. Its primary mechanism involves GPX4 utilizing GSH as a substrate to reduce lipid hydroperoxides (LOOH) to non-toxic lipid alcohols (LOH) through redox reactions, thereby limiting the propagation of lipid hydroperoxides within the membrane (Stockwell et al., 2017). Therefore, the activity of GSH and GPX4 is crucial. Studies have shown that indirectly or directly inhibiting GPX4 by depleting glutathione or blocking its activity can induce ferroptosis (Stockwell et al., 2017). And increasing exogenous GSH can eliminate ROS accumulation caused by iron deposition and lipid peroxidation, thus inhibiting cell death via ferroptosis (Sun et al., 2020). GSH is synthesized from glutamic acid (Glu), cysteine (Cys) and glycine (Gly). Cysteine and glutamic acid enter cells through the cystine/glutamate antiporter (system Xc-). Inhibition of system Xc-'s transport function, leading to reduced Cys and Glu exchange. It can hamper GSH synthesis and GPX4 activity, ultimately inducing ferroptosis (Sun et al., 2020). This highlights the close association between system Xc-'s activity, amino acid metabolism and ferroptosis. In experiments, the inhibition of GPX4 activity has been observed to induce ferroptosis. The system Xc-/GSH/GPX4 axis represents a pivotal mechanism for regulating ferroptosis, and all three pathways on this axis can serve as effective targets to modulate cellular ferroptosis (Shimada et al., 2016). We can inhibit ferroptosis by enhancing glutamate transport, promoting GSH synthesis, and increasing GPX4 activity, thereby bolstering the cell’s antioxidant capabilities.

#### 2.2.4 Other pathways of regulating ferroptosis

The p53 gene plays a pivotal role as a tumor suppressor in human physiology and is involved in various cellular processes such as proliferation, apoptosis and differentiation (Wu et al., 2019). Recent researches have indicated that p53 can modulate the pathways associated with ferroptosis (Ou et al., 2016). HUANG et al. discovered that p53 can downregulate the expression of SLC7A11, thereby inhibiting system Xc-, resulting in reduced cystine uptake and decreased GSH synthesis. This, in turn, further suppresses the activity of GSH-Peroxidase 4 (GSH-Px4), ultimately inducing ferroptotic cell death (Huang et al., 2018). CHU et al. identified a novel ferroptosis regulation pathway distinct from GPX4, where p53 can regulate ferroptosis with the assistance of ALOX12 (Chu et al., 2019). Studies have revealed that increased expression of heme oxygenase-1 (*HO-1*) leads to lipid peroxidation of cell membranes during the process of ferroptosis. *HO-1* serves as an essential enzyme in iron-dependent lipid peroxidation during ferroptosis. The *HO-1* pathway can regulate the process of ferroptosis (Kwon et al., 2015).

In general, ferroptosis involves multiple pathways such as iron metabolism, lipid metabolism, and amino acid metabolism, and can be regulated by multiple pathways, such as LOX, SystemXc-/GSH/GPX4, SLC7A11, P53/ALOX12, *HO-1*, etc. The downstream molecules of cell death caused by ferroptosis are not yet fully understood, but it has been determined to be closely associated with lipid peroxidation.

## 3 Iron metabolism mechanisms in intestinal cells and immune cells

The process of how red blood cells deliver iron to intestinal epithelial cells is not fully understood. Lu’s 1995 hypothesis suggests an active iron transport mechanism mediated by transferrin and its receptor at the basolateral side of intestinal cells under iron deficiency (Lu and Lin, 1995). This mechanism increases iron uptake from the intestine into the body and decreases intracellular iron content, facilitating iron entry from the intestinal lumen. Conversely, in iron excess, there is reduced iron uptake into the body, increased intracellular iron content, and decreased absorption, maintaining stable iron levels.

Subsequent studies validated this hypothesis, demonstrating that iron within intestinal cells can be stored as ferritin or exported to the bloodstream via the iron transporter protein ferroportin. The process of iron uptake by intestinal epithelial cells is influenced by inhibitors such as gastric acid, ascorbic acid, phytates, and polyphenols, with particular relevance to the activity of the iron transporter protein (Allocca et al., 2014). The expression of ferroportin is regulated by the peptide hormone hepcidin. Hepcidin (Krawiec and Pac-Kozuchowska, 2014), a 25-amino acid peptide primarily synthesized in hepatocytes, plays a crucial role in regulating systemic iron homeostasis. During inflammatory states, cytokines such as interleukin (IL)-6, IL-1, and IL-22 can activate hepcidin transcription. Elevated levels of hepcidin bind to ferroportin, causing its internalization and degradation within intestinal cells and macrophages. Consequently, iron becomes trapped within these cells, leading to iron deposition in intestinal epithelial cells and macrophages, and the occurrence of functional iron deficiency anemia. In states of iron deficiency or increased erythropoiesis, decreased hepcidin expression boosts iron absorption and release from macrophages.

These findings demonstrate the interplay of iron uptake, storage, and regulatory proteins in intestinal epithelial cells and macrophages, influenced by ferroportin and hepcidin activity. Inflammation triggers iron deposition in IEC and macrophages through ferroportin and hepcidin, ultimately leading to ferroptosis.

## 4 Mechanisms of ferroptosis in UC

### 4.1 Overview of ferroptosis in UC

The pathogenesis of UC is based on the damage to the intestinal mucosal barrier. The normal physiological function of the IEC is closely related to the intestinal mucosal barrier (Gilbert et al., 2012). IEC, derived from intestinal stem cells, includes goblet cells, enteroendocrine cells, which can secrete antimicrobial peptides and other substances to isolate the intestinal microbiota, thus constituting the intestinal mucosal barrier (Pinto and Clevers, 2005; van der Flier and Clevers, 2009). Abnormal death of IEC can disrupt the integrity of the intestinal mucosal barrier, leading to the occurrence of UC. So far, various cell death patterns, including apoptosis, necrosis, necroptosis, and autophagy, have been found to be involved in UC. However, the underlying mechanisms of UC cannot be fully explained by these classical cell death patterns (Wang et al., 2024).

Studies have observed that induction of H_2_O_2_ leads to the death of intestinal epithelial cell-6 (IEC-6), causing an increase in ferroptosis-related proteins (iron levels, GSH, GPX4) during the process of UC. The use of the ferroptosis inhibitor ferrostatin-1 can inhibit the death of IEC-6 cells (Huang et al., 2022). Experiments have detected an increase in the levels of ferroptosis-related markers (GPX4, ACSL4, and FTH1) during the development of DSS-induced UC in mice (Chen et al., 2020). The use of ferroptosis inhibitors ferrostatin-1 and liproxstatin-1 significantly alleviated weight loss and colon shortening in DSS-induced mice and significantly reduced the inflammatory index. Additionally, the expression of GPX4, ACSL4, and FTH1 was reversed to normal levels after treatment (Chen et al., 2020). This proves that ferroptosis induces IEC death, disrupts the intestinal mucosa, and triggers UC. And inhibiting ferroptosis can alleviate UC.

The evidence above demonstrates that inhibiting ferroptosis can alleviate UC, but the underlying mechanisms remain unclear. Therefore, this study will elucidate the reasons for ferroptosis occurrence and how it exacerbates UC by examining the uptake and absorption of iron by intestinal cells, the role of iron in the immune system and intestinal mucosal barrier.

The specific mechanism of ferroptosis-induced UC can be elucidated from the following three aspects. Upon external stimuli, inflammation occurs in the intestine, leading to iron deposition in IEC and macrophages. Iron deposition impairs the ability of macrophages to eliminate pathogens, weakening the body’s immune response and further exacerbating inflammation. This cascade of events induces ferroptosis within IEC and macrophages. When ferroptosis occurs, lipid peroxidation caused by ferroptosis will destroy the cell membrane of IEC, disintegrate IEC, making it unable to secrete mucus to protect the intestine, and disrupt the intestinal mucosal barrier (Cheng et al., 2023). At the same time, the released materials into the extracellular space will continuously stimulate macrophages to overproduce pro-inflammatory tumor necrosis factor-α (TNF-α) and other pro-inflammatory factors, encapsulating the already fragmented IEC for its excretion. This will promote epithelial cell apoptosis and further compromise the integrity of the intestinal barrier (Cao et al., 2013), leading to sustained inflammation in the intestine (Kaser et al., 2008). On the other hand, the antioxidant axis SystemXc-/GSH/GPX4 axis involved in the process of ferroptosis can eliminate the oxidative stress state in UC and alleviate UC. The occurrence of ferroptosis will deplete GSH and GPX4, causing cells to be in a state of high oxidative stress, promoting the development of UC (Huang et al., 2022). The mechanisms of ferroptosis in UC and the current status of drug targeting ferroptosis for treating UC will be described separately from the perspectives of iron deposition, lipid peroxidation and oxidative stress.

### 4.2 Iron deposition disrupts the immune system and aggravates UC

In 2004, Gasche proposed that a certain amount of iron is essential for lymphocyte development, but iron deposition in immune cells hinders basic immune functions and the host’s resistance to invading pathogens (Gasche et al., 2004). As previously discussed, normal iron uptake by cells relies on iron transport proteins. In cases where external factors such as intestinal dysbiosis trigger inflammation, the impact on the immune system includes: inflammation stimulates macrophages to reduce iron efflux function, leading to a loss of ability to kill intracellular pathogens.

The imbalance in iron homeostasis affects the cytokine activity of macrophages and cell-mediated immune response mechanisms. High iron loads diminish responsiveness to interferon-gamma (IFN-γ), resulting in reduced expression of anti-inflammatory factors like TNF-α and MHC class II antigens, leading to Th1/Th2 imbalance with weakened Th1 effector functions and increased production of Th2-mediated cytokines. Consequently, these macrophages lose their capacity to kill intracellular pathogens through the IFN-γ pathway (Gasche et al., 2004). The iron transport by macrophages significantly impacts the homeostasis of inflammatory responses, revealing a complex and contradictory relationship between iron and inflammation states. Regulating macrophage iron transport may offer new therapeutic avenues for such diseases.

### 4.3 Lipid peroxidation in UC

In 2000, Millar discovered that the use of iron chelators significantly reduced ROS in the colon tissues of UC patients, thereby improving clinical symptoms and endoscopic findings in UC (Millar et al., 2000). Further research has found that the use of iron chelators can reduce the production of lipotoxic ROS in IEC of UC mice, to prevent UC (Minaiyan et al., 2012). When there is abnormal iron metabolism, Fe^2+^ accumulates, and excess Fe^2+^ reacts with H_2_O_2_ in the cells to produce the Fenton reaction, generating -OH. -OH can oxidize lipids in IEC to form lipid peroxides (Li et al., 2015), leading to mitochondrial damage, cell membrane disintegration, causing IEC death, disrupting the intestinal mucosal barrier, and leading to the occurrence of UC.

As mentioned earlier, the main targets of lipid peroxidation are PUFAs. Magtanong et al., 2019 research has found that exogenous MUFAs can replace PUFAs, lower phospholipid levels containing oxidizable PUFAs, consume ROS, inhibit ROS accumulation on the membrane, thereby inhibiting ferroptosis. Studies have found that ω-3 PUFAs can alleviate mouse UC by competitively reducing arachidonic acid-derived leukotrienes, activating PPARγ, and promoting the synthesis of anti-inflammatory resolvins (Magisetty et al., 2023). This indicates that controlling the direction of lipid metabolism can be a way to target ferroptosis to regulate the progression of UC (Yokote et al., 2023).

It has been demonstrated that LOX catalyzes the production of lipid hydroperoxides and drives ferroptosis (Loscalzo, 2008; Hassannia et al., 2019). Experimental evidence has shown that 12/15-LOX can generate anti-inflammatory lipid mediators, catalyzing the protection of eosinophils against acute murine colitis (Masterson et al., 2015). Selective inhibitors of 5-LOX can alleviate the inflammatory state in the colon of UC patients (Staerk Laursen et al., 1990). Phosphatidylethanolamine binding protein 1(PEBP1)is a scaffold protein inhibitor of protein kinase cascades. It forms complexes with two isoforms of 15LOX: 15LOX1 and 15LOX2, and promotes the oxidation of PUFAs by 15-LOX to catalyze the ferroptosis process (Wenzel et al., 2017). Research has found that PEBP1-deficient mice are resistant to DSS- or TNBS-induced colitis and accelerate the recovery of intestinal mucosal damage (Lin et al., 2017). In summary, LOX plays an important role in catalyzing lipid peroxidation, ferroptosis, and promoting the development of UC. LOX inhibitors may serve as a pathway for inhibiting ferroptosis to treat UC.

In conclusion, ferroptosis accelerates the progression of UC through lipid peroxidation.So inhibiting ferroptosis can serve as a way to treat UC, involving key pathways of lipid metabolism such as LOX, PUFAs.

### 4.4 Oxidative stress and system Xc-/GSH/GPX4 in UC

Oxidative stress (OS) is considered a crucial pathogenic factor in UC. In the context of UC, oxidative stress is a pivotal contributor to the pathological processes, further exacerbating the condition by inflicting damage upon intestinal epithelial cells (IECs) through lipid, protein, and DNA injury (Holmes et al., 1998). This OS state arises from an excessive generation of ROS coupled with an insufficient capacity of the antioxidant system to counteract them. Numerous experimental studies have demonstrated a reduction in the levels of crucial antioxidant enzymes, such as GSH and GPX4, in UC mice and patients. Conversely, increasing the activity of GSH and GPX4 has been shown to significantly ameliorate colonic health and treat UC. It highlights the close association between the antioxidative mechanism and the development and management of UC (Wang et al., 2020). When ferroptosis occurs within IECs, it leads to an overproduction of ROS, depleting GSH levels and inhibiting GSH synthesis. Ultimately it impairs the normal functioning of the antioxidant axis. This results in elevated levels of myeloperoxidase (MPO) and malondialdehyde (MDA), accompanied by a reduction in superoxide dismutase (SOD) levels, inducing a state of heightened OS.

As previously discussed, the System Xc-/GSH/GPX4 axis represents a pivotal intracellular antioxidative mechanism. This axis effectively eliminates the surplus ROS generated during ferroptosis, alleviating oxidative stress and mitigating the heightened OS state in UC. Research indicates that pharmaceutical interventions can inhibit ferroptosis and ameliorate UC by promoting GSH synthesis. This underscores the therapeutic potential of enhancing the functionality of the antioxidant axis to treat UC targetting ferroptosis (Cheng et al., 2023). Research has found that butyrate can treat DSS-induced UC in mice. Its mechanism involves promoting *Nrf2*/GPX4 signaling, increasing GPX4 activity, inhibiting ferroptosis, and improving intestinal barrier integrity (Chen et al., 2023). Furin is a proprotein convertase that can treat UC, and it has been reported to promote *Nrf2*/GPX4 signaling, increase GPX4 activity, inhibit ferroptosis, and treat UC (Dong et al., 2021). Deferasirox is a synthetic iron chelator. Experimental evidence has shown that the use of deferasirox in treating DSS-induced UC in mice significantly improves the oxidative stress index, upregulates the expression of GPX-4 and FTH, inhibits ferroptosis, and treats UC (Wu et al., 2023).

In summary, drugs can upregulate the content of GSH and GPX4 through various signaling pathways, thereby treating UC by inhibiting ferroptosis and promoting antioxidation.

### 4.5 The impact of other regulatory factors of ferroptosis in UC

Research has revealed the influence of various factors on ferroptosis and its consequences in UC. It has been observed that NF-κBp65, through the inhibition of endoplasmic reticulum stress, plays a protective role in preventing ferroptosis in intestinal epithelial cells. This suggests that targeting the phosphorylation of NF-κBp65 to suppress ferroptosis represents a potential therapeutic target for UC management (Xu et al., 2020). Studies have demonstrated that vitamin D, by downregulating ACSL4, regulates the antioxidant axis, subsequently inhibiting ferroptosis and alleviating UC. Collectively, these findings indicate that pharmaceutical interventions can modulate ferroptosis through various pathways, thereby exerting a regulatory influence on the progression of UC. Research has found that the expression of *Nrf2* and *HO-1* increases in DSS-induced UC mice. Furthermore, ferrostatin-1 reversed the expression of *Nrf2* and *HO-1*, suggesting that ferritin inhibits iron overload through the *Nrf2*/*HO-1* pathway, thereby improving DSS-induced UC (Chen et al., 2020). This demonstrates that ferroptosis is a key factor in the treatment of UC, providing a new perspective for understanding the molecular mechanisms of UC and developing new treatment strategies.

In conclusion, ferroptosis can lead to UC by damaging IECs and stimulating macrophages to secrete pro-inflammatory factors, thereby damaging the intestinal mucosa. This process involves the mechanism of lipid peroxidation. Drugs can inhibit ferroptosis and treat UC by controlling lipid metabolism, seeking alternatives to PUFAs, or targeting proteins related to the LOXs family to control the process of lipid peroxidation. UC is also associated with the destruction of the antioxidant axis System Xc-/GSH/GPX4 during the process of ferroptosis. Ferroptosis depletes GSH and GPX4, leading to oxidative stress and causing UC. Drugs can promote the synthesis of GSH and GPX4 to inhibit ferroptosis and treat UC, involving proteins or pathways such as *Nrf2*/GPX4 and FTH. Thus, although the mechanism between ferroptosis and UC is not fully elucidated, it is closely related, and multiple pathways for drug targeting of ferroptosis to treat UC have been confirmed.

## 5 Correlation between UC risk factors and ferroptosis induction

### 5.1 Endoplasmic reticulum stress

Endoplasmic reticulum (ER) stress has been demonstrated to exacerbate intestinal inflammation and promote the development of UC (Park et al., 2019). Both lymphocytes and IECs inducing ER stress can lead to intestinal dysfunction, thereby resulting in or worsening inflammatory diarrhea. Protein kinase RP-like ER kinase (PERK) serves as the primary sensor for ER stress. Studies have shown that the use of PERK inhibitor CSK414 significantly reduces IEC death and improves experimental colitis. Moreover, specific loss of NF-κB p65 in IECs exacerbates DSS-induced murine UC via ER stress-mediated IEC death (Xu et al., 2020), indicating that IEC death is regulated through ER stress-mediated epithelial cell death. Further investigations revealed that phosphorylated nuclear factor κB (NF-κB) p65 interacts with its regulatory factor eIF2α to inhibit ER stress-mediated IEC death (Xu et al., 2020). It suggested that NF-κB p65 may be a potential therapeutic target for UC.

### 5.2 *Nrf2*/*HO-1* pathway

The *Nrf2* pathway has been proven to be a therapeutic pathway associated with UC and can inhibit ferroptosis and protect IECs through the *Nrf2*/GPx4 signaling pathway (Chen et al., 2020). Recent studies have shown that *Nrf2* is a key factor in inhibiting ferroptosis, yet excessive activation might lead to ferroptosis. On the one hand, Wu, 2021 found that Nrf2 and HO-1 are significantly upregulated in murine colitis, thus exerting anti-inflammatory and antioxidant effects. Wu treated DSS-induced UC mice with deferoxamine and found that it not only modulated the release of inflammatory factors but also enhanced the expression of Keap-1 and *HO-1*, related antioxidant proteins in the *Nrf2* pathway in colonic tissues to ameliorate inflammation. This suggests that the *Nrf2*/*HO-1* pathway may have a protective effect on the gut by inhibiting ferroptosis. But on the other hand, research by CHEN et al. found that Astragalus polysaccharides (APS) could prevent murine colitis and ferroptosis in human Caco-2 cells by inhibiting this signaling pathway.It suggested that ferroptosis may be regulated through the *Nrf2*/*HO-1* signaling pathway in DSS-induced UC (Chen et al., 2020). In the study, the expression of *Nrf2* and *HO-1* was upregulated in DSS-induced UC mice. However, the ferroptosis inhibitor Ferrostatin-1 reversed the expression of *Nrf2* and *HO-1*. Chen et al. speculated that ferroptosis may regulate DSS-induced UC through the *Nrf2*/*HO-1* signaling pathway. The specific mechanism of the *Nrf2*/*HO-1* signaling pathway in ferroptosis remains unclear and warrants further investigation.

### 5.3 Other programmed cell death

Programmed cell death (PCD) is a death mechanism involving complex signals that occur in an orderly manner and are regulated by genes. It mainly includes apoptosis, autophagy, ferroptosis, pyroptosis, and necroptosis. Apoptosis and autophagy have been proven as major forms of PCD and play important roles in the treatment of UC. Ferroptosis is also a type of PCD, distinguished from other PCD primarily by morphological and biological changes in cells. For example, apoptosis generally involves cell shrinkage, chromatin condensation, and formation of apoptotic bodies, accompanied by activation of the caspase protein family. Autophagy shows membrane structures resembling lipid bodies on the cytoplasmic membrane. These membrane structures progressively expand to form autophagosomes, engulfing cellular metabolites in the cytoplasm for degradation by hydrolytic enzymes in lysosomes (Chen et al., 2024). In contrast, cells undergoing ferroptosis exhibit mitochondrial shrinkage, reduced cristae, increased membrane density but with normal nuclear size morphologically, and biologically present iron deposition and iron-dependent accumulation of abundant lipid ROS. Protein and mRNA expression, as well as cellular morphology, detected in the treatment of UC by targeting different PCDs are distinct. By examining this information, ferroptosis can be distinguished from other PCDs during the investigation of drug mechanisms of action. Some traditional Chinese medicines can regulate ferroptosis for treating UC, as well as treat UC through autophagy and other mechanisms. For instance, Zhang et al. found that curcumin inhibits the expression of autophagy-related proteins Atg12, Beclin-1, and LC3II to treat UC (Zhang et al., 2018). However, curcumin can also inhibit ferroptosis for treating UC (Yang et al., 2022). This suggests potential crosstalk and interactions among various PCD types, which require further exploration.

## 6 The mechanism of TCM regulating ferroptosis in the treatment of UC

### 6.1 TCM’s understanding of UC and ferroptosis

TCM does not have a direct equivalent name for UC, but based on its characteristics such as abdominal pain, diarrhea, and bloody stools, it can be classified under categories like “intestinal disorders” or “dysentery” (Liu et al., 2021). The use of TCM against UC dates back to the pre-Qin period. In thousands of years of clinical practice, single and compound TCM have shown good efficacy in treating UC and improving patients’ clinical symptoms. Single herbs like “Huang Lian (Yan et al., 2020)” and “Qing Dai (Yang et al., 2023)” can all clear heat, stop diarrhea, regulate qi, and promote blood circulation. Herbal formulas such as “Bai Tou Weng Tang (Liu et al., 2023)” and “Huang Qin Tang (Jiang et al., 2023)” can also clear heat, promote diuresis, regulate qi, and activate blood circulation. Based on contemporary research, TCM has demonstrated anti-inflammatory, antioxidant, anti-apoptotic, and mucosal protective effects, presenting a multi-faceted approach to preventing UC (Wang et al., 2024). Clinical studies have indicated that the combination of traditional Chinese and Western medicine in the treatment of UC surpasses single-drug therapy. TCM has shown significant improvement in patient symptoms, enhancing quality of life, with minimal toxic side effects and low recurrence rates (Wang et al., 2024). According to TCM, “qi” is considered the foundation of human beings. Qi is the vital substance that constitutes and maintains the life activities of the human body, it is a constantly moving and extremely subtle material. The movement of “qi” determines whether the human body functions normally. If various factors lead to an abnormality in the movement of qi, causing stagnation of qi and blood, it will result in the accumulation of pathogenic factors. This is precisely the pathogenesis of UC. In the perspective of TCM, UC is primarily attributed to external damp-heat or internal dietary influences, leading to the accumulation of pathogenic factors in the intestines, stagnation of Qi and blood, and resulting in the formation of pathological products such as “damp-heat, heat-toxin, and blood stasis,” ultimately damaging the intestinal network and causing diarrhea and bloody stools. The manifestation of iron deposition and lipid peroxide accumulation in ferroptosis serves as a microcosmic representation of the stagnation of Qi and blood in the intestines, aligning with the traditional concept of “damp-heat, heat-toxin, and blood stasis” (Yang et al., 2022).

An increasing body of research in the field of TCM for the treatment of UC suggests that numerous herbs and formulations composed of TCM possess the capacity to modulate the upstream and downstream molecular factors associated with ferroptosis (Guo et al., 2024). This modulation serves to inhibit ferroptosis and ameliorate the condition of UC. TCM achieves this by influencing essential factors involved in ferroptosis, such as iron metabolism and the antioxidant axis, through multiple signaling pathways including *Nrf2*/*HO-1* and GSH/GPX4, etc. By doing so, TCM effectively suppresses the occurrence of ferroptosis, subsequently reducing the levels of ROS, alleviating oxidative stress, protecting intestinal mucosa and ultimately contributing to the therapeutic management of UC. Furthermore, TCM formulations, which often involve a combination of various TCM, leverage the synergy between these metabolites to achieve a holistic therapeutic effect. This approach signifies the organic integration of TCM principles with modern medicine. Therefore, this review aims to summarize the role of ferroptosis mediated by the active metabolites of various TCM and to provide a new perspective for the treatment of UC.

We searched the electronic databases PubMed, Web of Science, Embase, and China National Knowledge Infrastructure database (CNKI), Wanfang up to 18 March 2024 with the key words “ulcerative colitis” and “ferroptosis,” “ulcerative colitis” and “traditional Chinese medicine,” “TCM”, or “natural plant,” etc. The aim was to elucidate the intrinsic mechanisms of TCM targeting ferroptosis in the treatment of UC.

**FIGURE 2:**
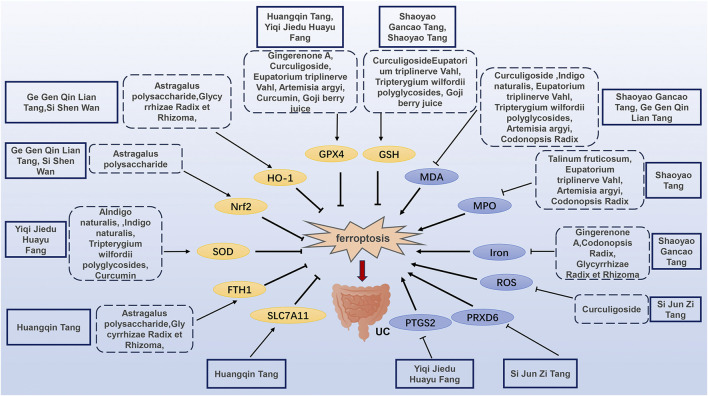
Mechanisms of TCM regulating ferroptosis in the treatment of UC.

The mechanism of TCM regulating ferroptosis in the treatment of UC is illustrated in Figure 2 and Table 1, and Table 2.

**TABLE 1:** Summary of TCM single herbs and extracts regulating ferroptosis in the treatment of UC.

Name	Source	Animal/cell model	Dosageand duration	Pharmacological actions	Reported targets	Reference
Curculigoside	*Curculigo orchioides* Gaertn	DSS-induced mice	50–100 mg/kg*7d	Attenuates oxidative stress	GPX4, GSH↑; MDA, ROS↓	Wang et al. (2020)
Talinum fruticosum	*Talinum fruticosum (*L*.) Juss*	DSS-induced rat	200 mg/kg*24 h	Attenuates oxidative stress	NO, MPO, TNF-α, IL-6, IL-1β↓	Oladele et al. (2023)
Curcumin	*Curcuma longa* L	DSS-induced mice	60 mg/kg*7d	Attenuates oxidative stress	MPO, MDA↓; SOD, GPX-Px↑	Yang et al. (2022)
Codonopsis Radix	*Codonopsis pilosula* (Franch.) Nannf	TNBS-induced rat	4.5–18 g/kg*7d	Attenuates oxidative stress	IL-18, L-6, IL-8, TNF-a, Fe^2+^, MDA, MPO↓; GPX4, GSH, SOD↑	Li et al. (2023)
Tripterygium wilfordii polyglycosides	*Tripterygium regelii Sprague & Takeda*	DSS-induced mice	3–12 mg/kg*14d	Reduces lipid peroxidation	MDA, TNF-α, IL-1β↓; SOD, GSH↑	Zhou et al. (2017)
Indigo naturalis	*Isatis tinctoria subsp. tinctoria*	DSS-induced mice	200–800 mg/kg*10d	Ameliorates inflammation	MDA, TNF-α, IL-6, IL-1, IL-10↓; SOD↑	Ma et al. (2019)
Artemisia argyi	*Artemisia argyi* H.Lév. & Vaniot	RSL3-induced macrophages	10–40 μm*6 h	Ameliorates inflammation	GPX↑; LDH, MDA↓	Wu et al. (2022)
Goji berry juice	*Lycium barbarum* L	DSS-induced mice	20 mL/kg*30d	Ameliorates inflammation	GPX4, GSH↑; MDA, IL-1β, IL-6, IFN-γ, TNF-α↓	Liu et al. (2021)
Isofraxidin	*Sarcandra glabra (*Thunb.*) Nakai*	DSS-induced mice	20–80 mg/kg*7d	Regulates *Nrf2* pathway	*Nrf2*↑; IL-18, IL-1β, TNF-α↓	He et al. (2024)
Gingerenone A	*Zingiber officinale* Roscoe	DSS-induced mice	5–50 mg/kg*7d	Regulates *Nrf2*/GPX4 pathway	Fe^2+^, TNF-α, IL-1β, IL-6↓; GPX4↑	Chen et al. (2022)
Astragalus polysaccharide	*Astragalus mongholicus* Bunge	DSS-induced mice	100–300 mg/kg*15d	Regulates *Nrf2*/*HO-1* pathway	*Nrf2*, *HO-1*, FTH1, GSH-Px4↑	Chen et al. (2021)
Glycyrrhizae Radix et Rhizoma	*Glycyrrhiza glabra* L	LPS-induced Caco2 cell	1.6–8 g/mL*24 h	Regulates *Nrf2*/*HO-1* pathway	*Nrf2*, *HO-1*, FTH1, GSH-Px4↑; Fe^2+^↓	Kong et al. (2023)

**TABLE 2:** Summary of TCM herbal formulas regulating ferroptosis in the treatment of UC.

Name	Prescription source	Animal/cell model	Dosageand duration (d)	Pharmacological actions	Reported targets	Reference
Shaoyao Gancao Tang	*Paeonia lactiflora* Pall. and *Glycyrrhiza glabra* L	DSS-induced mice	14 g/kg*7	Reduces iron deposition	Fe^2+^, MDA↓; GSH↑	Hu et al. (2023)
Huangqin Tang	*Scutellaria baicalensis* Georgi*, Paeonia lactiflora* Pall.*, Ziziphus jujuba* Mill.*,* and *Glycyrrhiza glabra* L	DSS-induced mice	4.55–18.2 g/kg*7	Attenuates oxidative stress	GSH-Px4, FTH1, SLC7A11↑	Wu et al. (2021)
Shaoyao Tang	*Paeonia lactiflora* Pall.*, Areca catechu *L*., Rheum officinale *Baill.*, Scutellaria baicalensis* Georgi*, Coptis chinensis *Franch.*, Angelica sinensis* (Oliv.) Diels*, Neolitsea cassia *(L.) Kosterm.*, Glycyrrhiza glabra* L.*, Dolomiaea costus *(Falc.) Kasana & A.K.Pandey	TNBS-induced mice	1 mg/kg*5	Attenuates oxidative stress	GSH↑; MPO, TNF-α, IL-1β, IL-6↓	Li et al. (2022)
Yiqi Jiedu Huayu Fang	*Codonopsis pilosula* (Franch.) Nannf., *Atractylodes macrocephala* Koidz.*, Paeonia lactiflora* Pall.*, Dolomiaea costus* (Falc.) Kasana & A.K.Pandey*, Portulaca oleracea* L.*, Coix lacryma-jobi* L.*, Bletilla striata* (Thunb.) Rchb.f.*, Panax notoginseng* (Burkill) F.H.Chen*, Hordeum vulgare* L.*, Coptis chinensis* Franch., *and Phellodendron amurense Rupr*	UC patients	200 mL/d*14	Attenuates oxidative stress	PTGS2↓; SOD1, GPX4↑	Chen et al. (2021)
Si Jun Zi Tang	*Panax ginseng* C.A.Mey.*, Atractylodes macrocephala* Koidz.*, Smilax glabra* Roxb.*, Glycyrrhiza glabra* L	DSS-induced mice	3.13–12.48 g/kg*28	Attenuates oxidative stress	PRXD6, ROS↓	Ning (2022)
Li Zhong decoction	*Panax ginseng* C. A. Mey., Z*ingiber officinale* Rosc., *Atractylodes macrocephala* Koidz., and *Glycyrrhiza uralensis* Fisch	DSS-induced mice	1.82–5.46 g/kg*7	Attenuates oxidative stress	MDA↓; SOD, GSH, GPX4↑	Li et al. (2024)
Ge Gen Qin Lian Tang	*Pueraria montana* var. *lobata* (Willd.) *Maesen & S.M.Almeida ex Sanjappa & Predeep*, *Scutellaria baicalensis* Georgi, *Coptis chinensis* Franch., and *Glycyrrhiza glabra* L	DSS-induced rat	17 g/kg*7	Regulates *Nrf2*/*HO-1* pathway	*Nrf2*/*HO-1*↑; MPO, LPO, MDA↓	Lin et al. (2022)
Si Shen Wan	*Cullen corylifolium* (L.) Medik.*, Tetradium ruticarpum* (A.Juss.) T.G.Hartley*, Myristica fragrans* Houtt.*, Schisandra chinensis* (Turcz.) Baill	DSS-induced mice	20–40 g/kg*7	Regulates *Nrf2*/*HO-1* pathway	*Nrf2*, *HO-1*, *NQO-1*↑; IL-6, TNF-α, MDA, ROS↓	Zhang et al. (2021)

### 6.2 Mechanism of TCM single herbs and extracts regulating ferroptosis in the treatment of UC

#### 6.2.1 Curculigoside (CUR)

Curculigoside is a phenolic glycoside metabolite derived from *Curculigo orchioides* Gaertn. TCM believes that Xianmao has the effects of eliminating dampness, detoxification, tonifying the kidneys, and nourishing the liver (Ju et al., 2020). Modern pharmacological studies have shown that it has antioxidant and anti-inflammatory properties (Ding et al., 2016). Research indicates that Curculigoside promotes the transcription level of GPX4 in IEC-6 cells, enhances GSH synthesis and reduces the production of MDA and ROS. Through the above, Curculigoside inhibits ferroptosis and alleviates oxidative stress, thus mitigating DSS-induced murine UC (Wang et al., 2020). Both CUR and ferroptosis inhibitor Ferrostatin-1 show similar degrees of inhibition on ferroptosis. Future experiments should include more dose-response to investigate the targeting effect of CUR on ferroptosis.

#### 6.2.2 Talinum fruticosum

Talinum fruticosum extract refers to the phytochemicals obtained from the plant *Talinum fruticosum* (L.) *Juss.* through thorough grinding, followed by extraction with distilled water and subsequent air drying. And it is commonly used to treat fever, ulcers, asthma, and measles. Recent studies have found that Talinum fruticosum extract can be effective in treating inflammatory gastrointestinal diseases, although the exact mechanism is not yet clear. Research indicates that Talinum fruticosum extract can inhibit inflammation in UC rats induced by DSS. Its mechanism of action involves reducing levels of NO, MPO, TNF-α, IL-6, and IL-1β, decreasing oxidative stress, increasing antioxidant enzyme activity, and inhibiting ferroptosis (Oladele et al., 2023). Additionally, Talinum fruticosum is more effective in inhibiting inflammation compared to sulphasalazine, although more dose-response for drugs were not set, and the minimum active concentration of drugs remains undetermined, necessitating further investigation.

#### 6.2.3 Curcumin

Curcumin is an active polyphenol derived from the underground rhizomes of the medicinal plant *Curcuma longa* L. TCM believes that Jiang Huang can promote blood circulation, regulate qi, and alleviate pain. It can be used to treat abdominal pain, diarrhea, and bloody stools. (Zong et al., 2014). Modern pharmacology suggests that Curcumin exhibits potent anti-inflammatory, antioxidant, and anti-tumor pharmacological activities (Yin et al., 2022). Examination of colon tissues shows that curcumin significantly reduces MPO and MDA levels while increasing SOD and GSH-Px levels, indicating its ability to inhibit ferroptosis, regulate oxidative stress levels, and treat DSS-induced UC in mice (Yang et al., 2022). The results of the experiment comparing Curcumin with atorvastatin calcium tablets should include more dose-response in future experiments.

#### 6.2.4 Codonopsis Radix

Dang Shen is the dried root of *Codonopsis pilosula* (Franch.) Nannf., a plant in the Campanulaceae family (Li et al., 2023). TCM believes that Dang Shen can invigorate the spleen, benefit the lungs, nourish the blood, and promote salivation. It has been frequently used by ancient and modern physicians in the clinical treatment of UC (Li et al., 2023). Network pharmacology and experiments have shown that Dang Shen downregulates the PI3K/Akt pathway, effectively reducing levels of IL-18, IL-6, IL-8, TNF-α in the serum of TNBS-ethanol composite-induced rats. It decreases the levels of Fe^2+^, MDA, and MPO, while increasing the levels of GPX4, GSH, and SOD activity. These actions demonstrate antioxidant stress and inhibition of ferroptosis, which are beneficial for treating UC (Li et al., 2023).

#### 6.2.5 Tripterygium wilfordii polyglycosides (TWP)

Tripterygium wilfordii polyglycosides (TWP) are extracted from the plant *Tripterygium regelii Sprague & Takeda* and possess potent anti-inflammatory and immunomodulatory effects. They can inhibit the expression of various cytokines, including IL-1, IL-2, and IL-6, and are commonly used in the treatment of inflammatory diseases of the digestive system (Ma et al., 2019). Studies have found that different doses of TWP effectively treat UC in DSS-induced mice, with a decrease in NF-κB, IL-1, and TNF-α levels during the treatment process (Ma et al., 2019). However, the specific mechanism of action has not been fully elucidated. Research has explored the use of TWP in UC rat models induced by TNBS and ethanol enemas to investigate its therapeutic mechanisms. TWP exhibits a dose-dependent reduction in MDA, IL-1β, and TNF-α levels, an increase in SOD and GSH levels, inhibition of lipid peroxidation, suppression of ferroptosis, promotion of colonic mucosal healing, and treatment of UC (Zhou et al., 2017). Comparatively, TWP has a similar therapeutic effect on UC as azathioprine tablets, yet its minimum active dose-response remains undetermined, warranting further exploration.

#### 6.2.6 Indigo naturalis

Indigo naturalis, derived from the plant *Isatis tinctoria subsp. tinctoria*, is an indole derivativ. TCM believes that Qing Dai, with its salty and cold nature, can clear heat, remove fire, detoxify, and stop diarrhea. As mentioned in “Ben Jing Fang Yuan,” it is said to “disperse depressed fire, treat warm toxins causing rashes, and treat severe *postpartum* fever and persistent diarrhea” (Jiang et al., 2019). Topical and oral application of indigo naturalis is used to treat various inflammatory diseases and skin conditions, including psoriasis (Deng et al., 2013). Recent studies have identified its significant role in UC. It can downregulate ferroptosis to decrease MDA, TNF-α, IL-6, IL-1, IL-10 levels and increase SOD levels, thus promoting mucosal healing and inhibiting DSS-induced murine UC (Ma et al., 2019). Experimentation revealed similar effects between Indigo naturalis and positive control group sulphasalazine, requiring further exploration.

#### 6.2.7 Artemisia argyi

Artemisia argyi oil is extracted from the plant *Artemisia argyi* H.Lév. & Vaniot and contains a rich array of chemical metabolites, including terpenoids and ketones. In TCM, Artemisia argyi leaves are believed to have properties that warm the middle and dispel cold, and are utilized in the treatment of various diseases, including colitis, arthritis, and dysmenorrhea. Modern medicine acknowledges the potential of this herb to suppress the release of pro-inflammatory mediators, such as nitric oxide, reactive oxygen species, TNF-α, IL-6, and IFN-β (Liu et al., 2021). The metabolite β-caryophyllene in the essential oil has been verified to reduce macrophage ferroptosis and inflammation in experimental colitis (Wu et al., 2022). β-Caryophyllene is a bicyclic sesquiterpene type-flavoring and fragrance material. Experimental findings indicate that β-caryophyllene activates CB2R, downregulates lipid peroxidation induced by RSL3, increases GPX4 activity, lowers LDH release and MDA content induced by RSL3 in macrophages, and inhibits macrophage ferroptosis (Wu et al., 2022). In future experimental designs, *in vivo* experiments should be conducted using animal models to continue exploring efficacy and mechanisms.

#### 6.2.8 Goji berry juice

*Lycium barbarum* L. is commonly known as goji berry, is a TCM and functional food. TCM believes that Gou Qi have the effects of nourishing the liver and kidneys, and gently tonifying deficiencies. They can be used to treat abdominal pain and diarrhea (Shen et al., 2015). Studies have demonstrated that goji berry can alleviate symptoms in murine UC and inhibit the expression of inflammatory factors such as IL-6, IL-1β, and TNF-α. Goji berry juice can increase anti-inflammatory cytokines, GSH levels, and reduces MPO levels, possibly related to ferroptosis (Liu et al., 2021). Future experiments should include more dose-response of Goji berry juice and shorten the experimental duration for further exploration.

#### 6.2.9 Isofraxidin

Isofraxidin is a coumarin compound extracted from the TCM *Sarcandra glabra* (Thunb.) *Nakai*. TCM attributes it with the effects of clearing heat, cooling blood, while modern pharmacology suggests anti-inflammatory and antioxidant properties. Studies have shown that Isofraxidin can inhibit ferroptosis by upregulating *Nrf2* and reducing ROS levels. Isofraxidin improved disease activity index weight, decreased secretion levels of inflammatory factors IL-18, IL-1β, and TNF-α in mice and cells, thereby alleviating DSS-induced UC in mice (He et al., 2024).

#### 6.2.10 Gingerenone A

Gingerenone A is a ketone extracted from *Zingiber officinale* Roscoe. According to TCM, Ginger, which has a warm nature, is believed to warm the middle and tonify the spleen to stop diarrhea (Yang et al., 2023). The anticancer, antioxidant, and antibacterial effects of Gingerenone A have been confirmed, and it is commonly used in the treatment of gastrointestinal diseases (Hughes et al., 2021).In recent research, it has been shown that Gingerenone A can significantly ameliorate clinical symptoms such as purulent bloody stools induced by UC, effectively enhancing intestinal barrier function (Suk et al., 2017). By assessing the activity of Fe^2+^, TNF-α, IL-1β, IL-6, GPX4 and *Nrf2*, it’s found that Gingerenone A can downregulate Fe^2+^, TNF-α, IL-1β, and IL-6 while upregulating GPX4 expression. The mechanism is Gingerenone A can regulate iron metabolism pathways by activating the *Nrf2*/GPX4 pathway, thereby controlling and alleviating ferroptosis in DSS-induced murine UC, ultimately mitigating UC (Chen et al., 2022). High doses of Gingerenone A have a greater therapeutic effect on UC than 5-aminosalicylic acid.

#### 6.2.11 Astragalus polysaccharide (APS)

Astragalus polysaccharide is sourced from *Astragalus mongholicus* Bunge. TCM believes that Huang Qi can tonify qi, strengthen the spleen, raise yang, and stop prolonged diarrhea and dysentery (Dai, 2019), Modern pharmacological studies have shown that Astragalus polysaccharide is a natural bioactive compound with antimicrobial, anti-inflammatory and metabolic regulatory properties. It has been demonstrated that Astragalus polysaccharide exerts protective effects in DSS-induced murine colitis by inhibiting the activation of NF-κB and the NLRP3 inflammatory complex (Lv et al., 2017). Recent research has observed that Astragalus polysaccharide can suppress the *Nrf2*/*HO-1* pathway to upregulates *Nrf2*, *HO-1*, FTH1, and GSH-Px4, thereby inhibiting ferroptosis in murine IEC and offering a therapeutic solution for UC (Chen et al., 2021). This experiment lacked a drug control group, only using a blank as the control, which is a limitation. Future experiments should include appropriate drugs, such as 5-aminosalicylic acid, for comparison.

#### 6.2.12 Glycyrrhizae Radix et Rhizoma (GR)

Glycyrrhizae Radix et Rhizoma is the dried root and rhizome of the leguminous plant *Glycyrrhiza glabra* L. TCM believes that Gan Cao can tonify qi, strengthen the spleen, and alleviate urgency and pain. It is commonly used in the treatment of UC (Zhang et al., 2015). It has been shown to improve UC by modulating gut microbiota, exhibiting antioxidant and anti-inflammatory effects (Shi et al., 2022). Examination of the *Nrf2*/*HO-1* pathway and ferroptosis-related proteins reveals that after using Glycyrrhiza uralensis, GSH/GPX and SOD levels significantly increase in Caco2 cells *in vitro*, Fe^2+^ levels significantly decrease. And the protein expression levels of *Nrf2*, *HO-1*, FTH1, and GSH/GPX4 significantly rise. This suggests that Glycyrrhizae Radix et Rhizoma promotes the expression of the *Nrf2*/*HO-1* pathway and regulates iron metabolism. It can inhibit ferroptosis and alleviate lipid peroxidation to improve LPS-induced Caco2 cell UC (Kong et al., 2023). In future experiments, *in vivo* experiments should be conducted using animal models to further validate the role of Glycyrrhizae Radix et Rhizoma in UC.

Other metabolites such as Liquiritin, Protocatechuic acid, Luteolin, Baicailin, Berberine, Evodiamine, Sinomenine, Tetrandrine, Oxynatrine, resveratrol, Crocin, Geniposide, and Lobetyolin and extracts such as Dandelion Root polysaccharides and Dendrobium polysaccharides are also used in the treatment of UC (Guo et al., 2024). And this review focused on TCM formulas as detailed in Table 2.

### 6.3 Mechanism of TCM herbal formulas regulating ferroptosis in the treatment of UC

The Shaoyao Gancao Tang, consisting of Bai Shao (*Paeonia lactiflora Pall.*) and Zhi Gancao (*G. glabra* L.), is a classic formula originating from the “Shanghan Zabing Lun”. It is known for its ability to harmonize the liver and spleen and alleviate liver-related urgencies. Research has demonstrated its effectiveness in treating UC in DSS-induced mice. By observing iron levels, GSH content, and mitochondrial morphology, the formula was found to downregulate Fe^2+^ levels, reduce iron deposition, GSH consumption and MDA levels. It also lowered ferroptosis levels within the colon tissue, ultimately decreasing inflammation markers and histological damage, effectively treating UC (Hu et al., 2023).

Huangqin Tang, derived from the “Shanghan Zabing Lun”, is composed of Huang Qin (*Scutellaria baicalensis* Georgi), Shaoyao (*P. lactiflora* Pall.), Dazao (*Ziziphus jujuba* Mill.), and Gancao (*G. glabra* L.). This formula is known for its heat-clearing and liver-calming properties. The general mechanisms of Huangqin Tang in treating UC involve alleviating oxidative stress and regulating mitochondrial autophagy, etc., (Chen et al., 2016). Recent research has revealed that after treating DSS-induced mice with Huangqin Tang, there is an increase in GSH/GPX4, FTH1 protein, and SLC7A11 mRNA expression. This suggests that Huangqin Tang may intervene in the ferroptosis mechanism through the GSH/GPX4 pathway to treat UC (Wu et al., 2021).

Shaoyao Tang, consisting of Shaoyao (*P. lactiflora* Pall.), Binglang (*Areca catechu* L.), Dahuang (*Rheum officinale* Baill.), Huangqin (*S. baicalensis* Georgi), Huanglian (*Coptis chinensis* Franch.), Danggui (*Angelica sinensis* (Oliv.) Diels), Guan Gui (*Neolitsea cassia* (L.) Kosterm.), Gancao (*G. glabra* L.), and Muxiang (*Dolomiaea costus* (Falc.) Kasana & A.K.Pandey), is known for its heat-clearing, dampness-removing and blood-regulating effects. Studies have found that Shaoyao Tang can activate GPX4, downregulate MPO, TNF-α, IL-1β, IL-6, and inhibit ferroptosis in IECs. This helps to restore barrier function and alleviate TNBS-induced mouse UC (Li et al., 2022).

The Yiqi Jiedu Huayu Fang is an empirical formula composed of Dang Shen (*C. pilosula* (Franch.) Nannf.), Chao Baizhu (*Atractylodes macrocephala* Koidz.), Chishao (*P. lactiflora* Pall.), Baishao (*P. lactiflora* Pall.), Wei Muxiang (*D. costus* (Falc.) Kasana & A.K.Pandey), Machixian (*Portulaca oleracea* L.), Chao Yiyiren (*Coix lacryma-jobi* L.), Baiji (*Bletilla striata* (Thunb.) Rchb.f.), Sanqi Powder (*Panax notoginseng* (Burkill) F.H.Chen), Chao Guya (*Hordeum vulgare* L.), Huanglian (*C. chinensis* Franch.), and Huangbai (*Phellodendron amurense Rupr.*). This formula is effective in downregulating UC-related proteins like PTGS2 while upregulating SOD1, GPX4, reducing ferroptosis, and inhibiting oxidative stress, thus treating UC (Chen et al., 2021).

The Si Jun Zi Tang, originating from the “Taiping Huimin Hejiju Fang,” consists of Renshen (*Panax ginseng* C.A.Mey.), Baizhu (*A. macrocephala* Koidz.), Fuling (*Smilax glabra* Roxb.), and Gancao (*G. glabra* L.). It serves as a fundamental formula for invigorating qi and tonifying the spleen. By measuring mitochondrial ROS levels and PRDX6 protein and mRNA expression, it has been found that Si Jun Zi Tang can lower ROS levels and PRDX6 expression, inhibiting ferroptosis in colonic tissue macrophages, thereby improving DSS-induced mouse UC (Ning, 2022).

The Li Zhong decoction (LZD), originating from the “Shang Han Lun,” is composed of Renshen (*P. ginseng* C. A. Mey.), Ganjiang (Z*ingiber officinale* Rosc.), Baizhu (*A. macrocephala* Koidz.), and Gancao (*Glycyrrhiza uralensis* Fisch.). It has the efficacy of warming the middle energizer and invigorating the spleen. LZD is commonly used in the treatment of gastrointestinal diseases such as UC. Studies have found that LZD can reduce iron overload and MDA, increase SOD, GSH, GPX4 to inhibit oxidative stress and alleviate ferroptosis in DSS-induced mice, thereby treating UC (Li et al., 2024).

The Ge Gen Qin Lian Tang consists of Ge Gen (*Pueraria montanavar. lobata* (Willd.) *Maesen & S.M.Almeida ex Sanjappa & Predeep*), Huangqin (*S. baicalensis* Georgi), Huanglian (*C. chinensis* Franch.), and Gancao (*G. glabra* L.). Experimental studies have shown that Ge Gen Qin Lian Tang can upregulate *Nrf2* and *HO-1* proteins, inhibit ferroptosis, significantly reduce serum levels of MPO, LPO, and MDA, improve colonic tissue pathological damage and structural integrity in UC rats, thereby treating UC (Lin et al., 2022).

The Si Shen Wan composed of Buguzhi (*Cullen corylifolium* (L.) Medik.), Wuzhuyu (*Tetradium ruticarpum* (A.Juss.) T.G.Hartley), Roudoukou (*Myristica fragrans* Houtt.), and Wuweizi (*Schisandra chinensis* (Turcz.) Baill.), is known for its kidney-warming, cold-dispelling, and intestine-binding properties. The formula can activate the *Nrf2*/*HO-1* pathway, upregulating *Nrf2*, *HO-1*, *NQO-1*, while downregulating ferroptosis-related proteins like IL-6, TNF-α, MDA, ROS levels, inhibiting ferroptosis and treating DSS-induced UC in mice (Zhang et al., 2021).

In summary, TCM extracts, single herbs and formulas can significantly downregulate iron-related proteins, such as Fe^2+^, MDA and ROS, as well as inflammatory factors like IL-6 and TNF-α. Simultaneously, they can upregulate antioxidant-related proteins like GSH, GPX4, and SOD, effectively blocking the progression of ferroptosis. TCM can target and regulate the upstream and downstream molecules of ferroptosis in the development of UC. This helps improve intestinal mucosa by reducing oxidative stress, protecting the mucosal barrier, and alleviating inflammation, ultimately treating UC. The mechanisms of TCM single herbs and extracts are more straightforward compared to formulations. This indicates that targeting ferroptosis with TCM could serve as a novel approach for treating UC. Future research should integrate traditional Chinese medical practices with modern medical studies to explore additional pathways between ferroptosis and UC.

While the experimental studies mentioned above have demonstrated significant therapeutic effects of TCM targeting ferroptosis in treating UC, there are still several limitations in the reviewed data that need to be discussed and improved upon. Firstly, TCM comprises various metabolites, and different extraction methods yield different components. For example, in the case of Goji berry juice mentioned above, it is unclear which metabolite is responsible for its effects. Secondly, there are issues with the comprehensiveness of the study designs. Some experiments only include fewer than three dosages of the active agents, such as Goji berry juice, Curcumin, and Curculigoside A. Rigorous pharmacological experiments should include experiments with multiple dose-response. Additionally, the duration of treatment is not standardized, and the control groups are not uniform, with some experiments only utilizing *in vitro* studies. Future research needs to involve stricter control groups and comparison experiments with different drugs such as 5-aminosalicylic acid, azathioprine and mesalazine. Furthermore, a variety of extraction methods and experimental models, including *in vivo* experiments, combined *in vitro* experiments, and clinical pharmacological experiments, should be utilized for further exploration. This will enable a comprehensive investigation into whether TCM targets ferroptosis, its interaction with other risk factors for exacerbating UC, and the types of metabolites involved. Further discussion is required to address the aforementioned issues.

## 7 Summary and prospects

Ferroptosis, a novel programmed cell death mechanism, participates in IEC death through various pathways, ultimately inducing UC, representing a crucial pathological mechanism of UC. The regulation of ferroptosis presents a potential therapeutic option for UC. Due to its natural origin and rare side effects, TCM has recently garnered increased attention and may therefore serve as an effective approach for ameliorating UC. In this review, we summarize several TCM extracts and single herbs, targeting ferroptosis in UC, along with an overview of their mechanisms and targets. These include *Nrf2*/GPX4, *Nrf2*/*HO-1*, FTH1, SLC7A11, etc. TCM formulas modulate ferroptosis through various signaling pathways, including *Nrf2*/*HO-1* and system Xc-GSH-GPX4. They have been utilized in the treatment of various experimental models of UC, such as DSS- induced and TNBS-induced UC, offering new perspectives and directions for the use of TCM in the treatment of UC.

In this review, we summarize several TCM herbs, metabolites, and prescriptions targeting ferroptosis in UC. It should be mentioned that TCM, especially formulas, have multiple ingredients/components, exerting multiple effects against UC, such as immune modulation, gut microbiota hemostasis maintenance, protection of intestinal barrier integrity (tight junctions), etc. Protection against ferroptosis and ferroptosis-mediated inflammation is only one of known mechanisms, which could play integrated roles together with other targets of TCM for UC prevention and treatments. We elucidate the connection between TCM, UC, and ferroptosis from the perspectives of traditional Chinese medicine and modern pharmacology. Traditional Chinese medicine categorizes UC into excess and deficiency syndromes, both characterized by the accumulation of “dampness-heat” and pathogenic factors. The iron deposition and lipid peroxidation associated with ferroptosis are microscopic manifestations of “dampness-heat”. The mentioned TCMs, such as Indigo naturalis, Curculigoside, and TCM prescriptions such as Shaoyao Gancao Tang, Huangqin Tang, and Shaoyao Tang, primarily eliminate the pathological products produced by ferroptosis through the methods of clearing heat, removing dampness, regulating qi, and blood to treat UC. Astragalus polysaccharide, Codonopsis Radix, Glycyrrhizae Radix et Rhizoma, and Sijunzi Tang mainly tonify qi, strengthen the spleen, warm the kidneys, and stop diarrhea, promoting the circulation of “qi” to facilitate the elimination of pathological products produced by ferroptosis, thus treating UC. We also review the mechanisms and signaling pathways of TCM targeting ferroptosis in the treatment of UC. These include Nrf2/GPX4, Nrf2/HO-1, FTH1, SLC7A11, Nrf2/HO-1, and the system Xc-GSH-GPX4. They have been used to treat various experimental models of UC induced by DSS, TNBS, etc., providing new insights and directions for TCM in the treatment of UC. This also demonstrates the close integration of TCM with modern pharmacology.

TCM exhibits tremendous potential in treating UC by regulating ferroptosis. However, further research is required before the clinical application of the aforementioned TCM single herbs, compounds, and formulas. Currently, the following issues need to be addressed in future research: 1) Due to the diagnosis of ferroptosis primarily relying on observations of mitochondrial morphology, iron content, ROS, MDA, and GPX4 expression, there is no gold standard for diagnosing ferroptosis, and its mechanism remains incompletely elucidated. 2) Research on the relationship between ferroptosis and UC is relatively superficial, and the related mechanisms of ferroptosis-induced UC and the pathways through which TCM acts on the upstream and downstream molecules of ferroptosis are not fully understood. Although ferroptosis exhibits varying degrees of interference with other regulated cell death mechanisms, such as apoptosis, autophagy, and necroptosis, uncertainties remain regarding how it mediates this network of multiple regulated cell death mechanisms. 3) Exploration of TCM formulas that regulate ferroptosis in UC is limited. Current research is focused on animal and cell models lacking support from clinical trial data, which poses a certain distance from clinical translation.

To address the above issues in future research, the following steps should be taken: 1) Design experiments using gene-deficient mice related to the proteins required at each stage of ferroptosis to thoroughly explore the mechanism of ferroptosis, providing more comprehensive data support for the treatment of UC using ferroptosis. 2) Further explore the mechanism of ferroptosis in UC, inhibit other activation conditions of ferroptosis, such as targeted regulation of the conversion between Fe^2+^ and Fe^3+^, or explore how to block the encounter of intracellular hydrogen peroxide and Fe^2+^, and design experiments to verify hypotheses. 3) Design clinical trials to perform biopsies of colon tissues from UC patients taking drugs that inhibit ferroptosis and track related pathway proteins to conduct a more in-depth exploration.

In conclusion, ferroptosis is an important mechanism involved in the pathogenesis of UC, and TCM demonstrate potential in regulating ferroptosis in the treatment of UC. However, research on the mechanisms of TCM, UC, and ferroptosis is limited. Further research is needed to provide high-quality evidence to translate research findings into clinical practice and offer new insights into the treatment strategies for UC.

## Abbreviations

FTH1, Ferritin Heavy Chain 1; GLU, Glutamate; GPX4, Glutathione peroxidase 4, GSH; Glutathion; GSH-Px, Glutathione peroxidase; HO-1, Heme oxygenase; H2O2, Hydrogen peroxide; IL-6, Interleukin-6; IL-18, Interleukin-18; IL-1β, Interleukin-1β; IFN-γ, Interferon-gamma; MDA, Malondialdehyde; MPO, Myeloperoxidase; NF-κB, Nuclear factor kappa-B; Nrf2, Nuclear factor E2-related factor 2; ROS, Reactive oxygen species; SOD, Superoxide dismutase; TCM, Traditional Chinese medicine; TNF-α, Tumor necrosis factor alpha; UC, Ulcerative colitis.
